# Relationship between self-management behavior and family care among Chinese older adults hospitalized for stroke: the mediating role of chronic disease resource utilization

**DOI:** 10.3389/fmed.2025.1611587

**Published:** 2025-07-09

**Authors:** Zhangyi Wang, Fen Zhu, Juan Zuo, Li Yang, Feng Lu, Li Chen, Liping Li, Xiaochun Tang, Tingrui Wang, Junbo Wu, Weimin Xie, Yating Zhan, Jiaofeng Peng, Yan Wang, Jie Yang, Yue Zhu, Zhenfa Li, Huilong Shen, Jun Yin

**Affiliations:** ^1^Nursing Department, Affiliated Hengyang Hospital of Hunan Normal University & Hengyang Central Hospital, Hengyang, China; ^2^Department of Hematology, The First Affiliated Hospital, Hengyang Medical School, University of South China, Hengyang, China; ^3^Gynecology Department, Chenzhou No. 1 People’s Hospital, Chenzhou, China; ^4^School of Nursing, Kiang Wu Nursing College of Macao, Hunan Normal University, Cotai, Macao SAR, China; ^5^School of Nursing, Guizhou Medical University, Guiyang, China; ^6^School of Nursing, Hengyang Medical School, University of South China, Hengyang, China; ^7^The Second Affiliated Hospital, Operating Room, Hengyang Medical School, University of South China, Hengyang, China; ^8^Nursing Department, Tianjin Academy of Traditional Chinese Medicine Affiliated Hospital, Tianjin, China

**Keywords:** older adults, hospitalized patients, stroke, self-management behavior, family care, chronic disease resource utilization, mediating role, China

## Abstract

**Background:**

The importance of self-management behavior has been widely acknowledged in global studies, demonstrating its effectiveness in improving patients’ emotional well-being, enhancing quality of life, preventing stroke recurrence, and reducing mortality and readmission rates. However, existing research indicates that self-management behavior among older adults hospitalized for stroke remains underdeveloped. Furthermore, limited studies have examined the correlations between self-management behavior, family care, and chronic disease resource utilization in this population.

**Objective:**

This study aims to investigate self-management behavior, family care, and chronic disease resource utilization among Chinese older adults hospitalized for stroke and to explore the relationships among these variables. Specifically, it examines the mediating role of chronic disease resource utilization between self-management behavior and family care.

**Methods:**

A cross-sectional correlational design followed the Strengthening the Reporting of Observational Studies in Epidemiology checklist for quality reporting. Between December 2023 and January 2025, a total of 627 Chinese older adults hospitalized for stroke were recruited from three tertiary grade-A hospitals in two cities in China. Data were collected using the Demographic Characteristics Questionnaire, the Stroke Patient Self-Management Behavior Assessment Scale, the Family APGAR Scale, and the Chronic Illness Resources Survey. Descriptive statistics, univariate analysis, correlation analysis, and process plug-in mediation effect analysis were applied to the data.

**Results:**

The total scores for self-management behavior, family care, and chronic disease resource utilization were (M = 165.94, SD = 51.26), (M = 5.51, SD = 1.65), and (M = 72.15, SD = 16.73), respectively. These scores indicate moderate levels of self-management behavior, family care, and chronic disease resource utilization. Self-management behavior was positively correlated with family care (*r* = 0.615, *p* < 0.01) and chronic disease resource utilization (*r* = 0.536, *p* < 0.01). Furthermore, chronic disease resource utilization partially mediated the relationship between self-management behavior and family care, accounting for 41.6% of the total effect.

**Conclusion:**

The self-management behavior, family care, and chronic disease resource utilization among Chinese older adults hospitalized for stroke were found to be at moderate levels, indicating a need for improvement. Family care directly affects self-management behavior and indirectly affects it through chronic disease resource utilization. Healthcare professionals should focus on enhancing health education and providing psychological support for stroke patients to improve family care, alleviate fears regarding disease progression, and ultimately promote better self-management behavior.

## 1 Introduction

Stroke, or cerebrovascular accident, occurs primarily when the blood vessels in the brain rupture or become blocked, leading to damage to brain tissues. It encompasses both hemorrhagic and ischemic strokes (1). Stroke is characterized by a high incidence, mortality, and disability rate (2). Projections of Zhang et al. (3) suggest that by 2030, the incidence of cerebrovascular diseases in China is expected to increase by approximately 50% compared to 2010. The severe disability caused by stroke results in a loss of patient’s ability to perform daily activities and work, placing a significant economic and psychological burden on their families and society while also affecting their physical and psychological health and overall quality of life. Due to economic and medical limitations, most stroke patients, after the acute phase, return to their families and society but continue to need long-term self-management—including disease monitoring and rehabilitation—to support recovery and prevent recurrence. Numerous studies indicate that self-management behavior is closely associated with the incidence, hospitalization rates, and mortality of stroke patients.

Lazarus’ stress and coping theory (4) suggest that stressors primarily drive individuals’ emotional, cognitive, and behavioral responses through cognitive appraisal and coping processes. This relationship underscores the continuity between support, cognition, coping styles, and adaptive outcomes. Self-management behavior refers to health-promoting actions in which patients manage and improve their physical health through self-monitoring, manage disease symptoms, and play an active role in disease treatment (5). Effective self-management behavior improves patients’ negative emotions, enhances quality of life, prevents stroke recurrence, and reduces mortality and readmission rates (6).

### 1.1 Literature review

#### 1.1.1 Family care as a predictor

Family care, defined as satisfaction with family functioning manifested through members’ support and concern, is a vital dimension of social support that plays a positive role in coping with life challenges (7). Within the Chinese cultural context, deeply influenced by Confucian values, family care extends beyond mere instrumental support. It embodies core tenets such as “filial piety” and familial responsibility, where caring for ill members is often perceived as a moral obligation and a natural expression of kinship solidarity (8, 9). Research consistently demonstrates a close relationship between family care and patients’ self-management behavior. For stroke patients, who frequently require assistance due to physical dysfunction, support from family, friends, and society becomes crucial for effective condition management. This support network, particularly the family unit, often operates within established hierarchical structures. The involvement and expectations of elders or key decision-makers within the family can significantly shape the nature and acceptance of care provided, influencing the patient’s adherence and self-management strategies (10).

Family care provides a robust psychological foundation. A harmonious and supportive family environment not only facilitates practical nursing, medication adherence, and dietary management but also alleviates negative emotions like anxiety and depression, thereby enabling the adoption of healthier behaviors and lifestyles (11, 12). The emphasis on family harmony in Confucianism further reinforces the positive emotional climate fostered by effective family care. Studies by Tong (13) and Cui et al. (14), conducted within Chinese populations, found a strong positive influence of family care on patients’ self-management behavior, indicating that higher levels of family care correlate with higher levels of self-management. Du et al. (15) further corroborated this positive relationship in 2022. Moreover, prior research (16, 17) indicates that family care can effectively mitigate anxiety and depression symptoms, improve cognitive function, and enhance health-related quality of life. Given that negative emotions are potent predictors of self-management awareness and behavior (18), the emotional buffering role of culturally embedded family care is particularly significant. However, the precise mechanisms underlying the impact of family care on self-management behavior, especially how culturally specific factors like filial piety norms, family hierarchy dynamics, and collective responsibility modulate this relationship, remain less clear and warrant further investigation.

#### 1.1.2 Chronic disease resource utilization as a predictor

Chronic disease resource utilization refers to how individuals access and utilize various social resources to support disease management (19). These resources can be broadly categorized into formal resources (e.g., healthcare institutions, government programs, professional services) and informal resources (e.g., family, friends, neighbors, community groups, religious organizations) (20). The former are typically structured, professional, and system-based, while the latter are often unstructured, relational, and arise from personal networks. Research by Halliday et al. (21) suggests that positive coping styles can enhance patient’ self-management behavior, and chronic disease resource utilization, encompassing both formal and informal types, can further support this behavior as a form of coping. This behavior reflects physical and mental conditions and shapes disease experiences.

The reliance on and accessibility of these resource types exhibit significant differences between urban and rural settings in China. Currently, China’s primary healthcare system faces limitations, particularly in rural areas, resulting in suboptimal access to and use of formal institutional resources by patients (22). Consequently, and compounded by the influence of traditional family values, patients across China, but especially in rural contexts where formal services may be scarcer or less accessible, tend to rely more heavily on informal resources, primarily family and friends, for support. This reliance often occurs at the expense of utilizing broader community-based resources available in neighboring communities, even when they exist in urban areas. Wang’s et al. (23) research in 2020 on inpatients with coronary heart disease indicates that social support, a key component of informal resources, is closely related to self-management behavior. Chronic diseases, with their prolonged course, significantly impact the social functioning of patients, and social support is recognized as a vital resource to help patients manage their diseases, alleviating stress and improving compliance (24). Social support is also considered an important external factor influencing the development and maintenance of healthy behaviors in patients. Medical professionals should guide patients to effectively utilize available social support resources (both formal and informal) and improve their management skills. However, the specific mechanisms through which the utilization patterns of these distinct formal and informal resources affect self-management behavior, particularly considering the urban-rural divide, remain to be fully elucidated.

### 1.2 Theoretical framework

**Figure 1:**
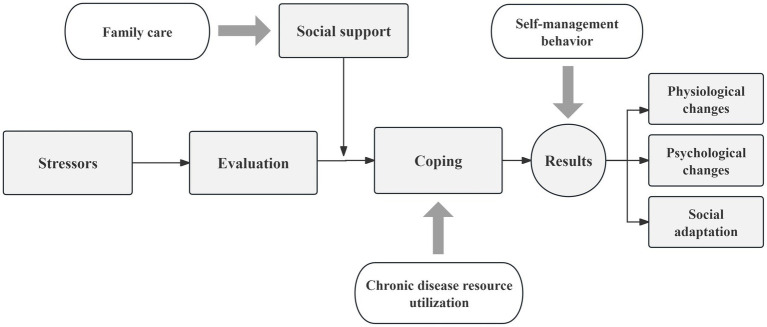
The theoretical diagram of Lazarus’s Stress and Coping Theory.

This study employs Lazarus’s stress and coping theory, developed by American psychologists Lazarus and Folkman (4). According to this theory, the stress process involves four aspects: cognitive evaluation of potential stressors, the stressors themselves, stress responses, and coping mechanisms. The occurrence of a stress response depends on both the cognitive assessment and the coping process. Cognitive evaluation includes primary evaluation, secondary evaluation, and re-evaluation. Lazarus proposed that coping can be understood from three perspectives: (1) as a style or quality, (2) as a resource, and (3) as a process. Stress responses result from interactions between individuals and their environment, manifesting as cognitive, emotional, and behavioral reactions. Stressors induce emotional, cognitive, and behavioral responses through cognitive evaluation and coping. In this study, family care is viewed as an integral part of social support, while the utilization of chronic disease resources represents a coping style employed by patients. These two factors work together to produce the outcome—self-management, which encompasses physiological and psychological aspects. The theoretical diagram of Lazarus’s stress and coping theory is shown in Figure 1.

**Figure 2:**
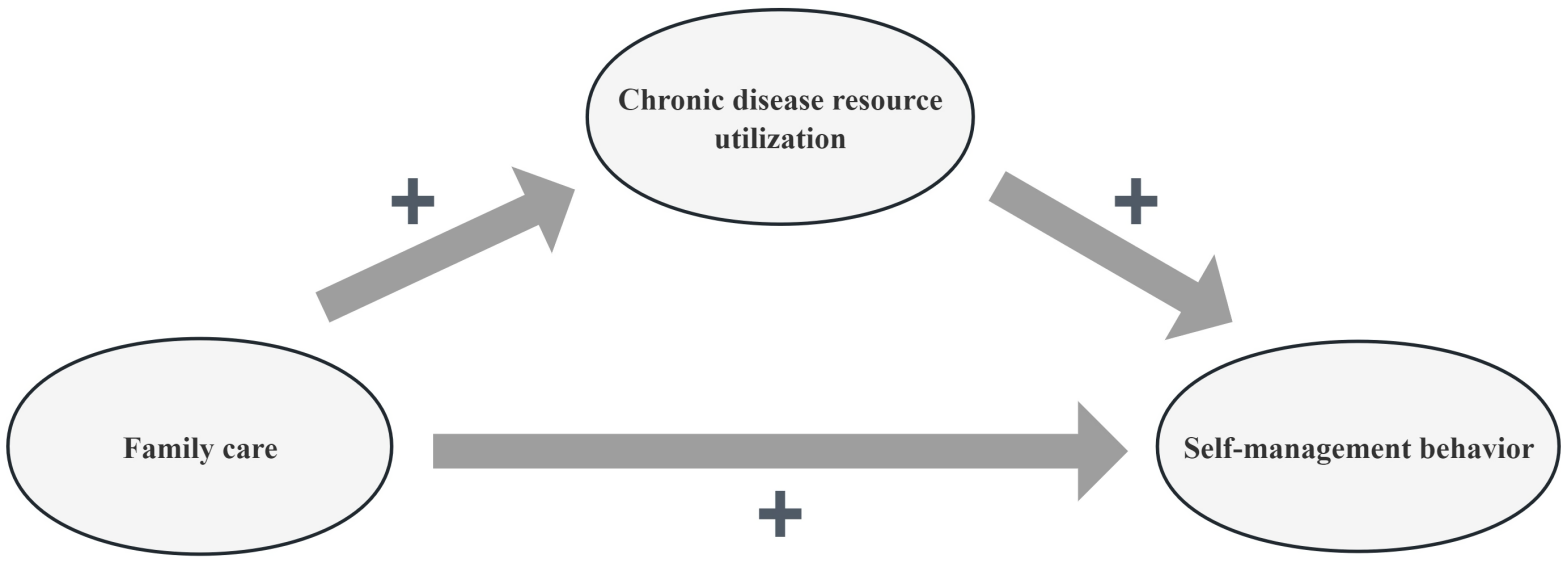
The conceptual framework of self-management behavior, family care, and chronic disease resource utilization of the study.

In summary, family care and chronic disease resource utilization may positively predict self-management behavior among Chinese older adults hospitalized for stroke. Moreover, chronic disease resource utilization may mediate between self-management behavior and family care. However, the mechanisms and relationships between self-management behavior, family care, and chronic disease resource utilization remain underexplored. Thus, further research is needed to clarify the connections and mediating roles of these factors. Based on the literature review and the theoretical framework, the conceptual model for this study is presented in Figure 2.

## 2 Objectives

The objectives of this study are to examine what is the relationship between self-management behavior, family care, and chronic disease resource utilization among Chinese older adults hospitalized for stroke.

## 3 Methods

### 3.1 Study design and setting

This study employed a descriptive cross-sectional design and was conducted in China. It adhered to the STROBE (Strengthening the Reporting of Observational Studies in Epidemiology) guidelines to ensure accurate, transparent, and comprehensive reporting.

### 3.2 Participants and sample

A convenience sampling method was used to recruit older Chinese adults hospitalized for stroke from three tertiary grade-A hospitals located in two cities in China. Recruitment occurred between December 2023 and January 2025. The inclusion and exclusion criteria for participants are as follows: inclusion criteria: (1) age ≥18 years; (2) patients in the recovery phase with stable conditions for more than 2 weeks; (3) ability to understand and complete the questionnaire and communicate with researchers; (4) voluntary participation. Exclusion criteria: patients with complex medical conditions, such as renal failure or mental illness, that would interfere with participation. Informed consent was obtained from all patients who voluntarily agreed to participate.

Based on G*Power 3.1.9.7 (25) calculations for *F*-tests (linear multiple regression: fixed model, *R*^2^ deviation from zero), assuming a medium effect size (*f*^2^ = 0.15), *α* = 0.05, and power = 0.95, the minimum required sample size was 107. A medium effect size was chosen based on prior literature reporting average correlation coefficients of approximately 0.16 among key variables (14, 15, 23). A total of 627 participants were ultimately recruited to enhance statistical power, account for potential missing data, and improve the generalizability of findings.

### 3.3 Measurements and variables

#### 3.3.1 The demographic characteristics questionnaire

The researchers developed the Demographic Characteristics Questionnaire based on relevant literature. It includes 17 items that capture basic demographic information, such as age, gender, nationality, employment status, marital status, education level, place of residence, primary caregiver, residence status, monthly income per capita, medical payment methods, stroke type, duration of illness, number of comorbidities, number of stroke occurrences, presence of functional impairment, and activities of daily living.

#### 3.3.2 The Stroke Patient Self-Management Behavior Assessment Scale

The Stroke Patient Self-Management Behavior Assessment Scale (SPSBAS) was developed by Wang (26) to assess self-management behavior in stroke patients. It consists of 50 items across 7 dimensions: “disease management” (11 items), “safe medication management” (5 items), “diet management” (8 items), “daily living management” (8 items), “emotional management” (5 items), “social and interpersonal management” (6 items), and “rehabilitation exercise management” (7 items). Responses are scored using a Likert scale, ranging from 1 (no) to 5 (always), with scores ranging from 50 to 250 points, scores of <125, 125–185, and >185, respectively, represent low, middle and high level of self-management behavior. Higher scores indicate better self-management behavior. The Cronbach’s *α* for the original scale was reported as 0.835; in this study, it was calculated as 0.918, indicating strong reliability.

#### 3.3.3 The Family APGAR Scale

The Family APGAR Scale (F-APGARS) was developed by Smiketen (27) and introduced to China by Lv et al. (28) in 1995 to assess family care in chronic disease patients. It consists of 5 items across 5 dimensions: “adaptation,” “partnership,” “growth,” “affection,” and “respect.” Responses are rated on a Likert scale from 0 (hardly ever) to 2 (often), with total scores ranging from 0 to 10 points. Higher scores indicate better family care. Scores of <3, 3–7, and >7, respectively, represent low, middle and high level of family care. The Cronbach’s *α* for the original scale was reported as 0.886; in this study, it was calculated as 0.915, indicating strong reliability.

#### 3.3.4 The Chronic Illness Resources Survey

The Chronic Illness Resources Survey (CIRS) was developed by Glasgow et al. (29) to assess chronic disease resource utilization in chronic disease patients. It consists of 22 items across 7 dimensions: “healthcare team” (3 items), “family and friends” (3 items), “personal coping” (3 items), “community resources” (4 items), “media and policy” (3 items), “organizational support” (3 items), and “work environment” (3 items). Responses are scored on a 5-point Likert scale, ranging from 1 (never) to 5 (always). The total score ranges from 22 to 110 points, with higher scores indicating greater chronic disease resource utilization. Scores of <40, 40–80, and >80, respectively, represent low, middle and high level of chronic disease resource utilization. The Cronbach’s *α* for the original scale was reported as 0.907, and in this study, it was calculated as 0.925, indicating strong reliability.

### 3.4 Data collection

Data collection was conducted between December 2023 and January 2025 across eight hospitals. After obtaining institutional approval and informed consent, participants were recruited with the assistance of head nurses. Face-to-face questionnaire administration ensured completeness; each survey took approximately 15–20 min. A total of 643 questionnaires were distributed, of which 627 were valid, yielding a response rate of 97.5%.

### 3.5 Statistical analysis

Data were analyzed using Epidata 3.1 and IBM SPSS 21.0. Descriptive statistics (frequencies, percentages, means ± SD) were computed. Group comparisons for normally distributed variables were conducted using independent-sample *t*-tests or one-way ANOVA; for non-normally distributed data, the Mann–Whitney *U* or Kruskal–Wallis tests were used. Pearson correlation coefficients were calculated to assess relationships among self-management behavior, family care, and chronic disease resource utilization. Mediation analysis was performed using the PROCESS macro (Model 4). Statistical significance was set at *p* < 0.05 (two-tailed).

### 3.6 Ethics considerations

This study was approved by the Ethics Committee of Hengyang Central Hospital (No. 2023-036-27). Written informed consent was obtained from all participants. The study adhered to the Declaration of Helsinki and relevant Chinese regulations. All responses were anonymous and used solely for academic purposes. Participants were informed of their right to withdraw at any time without penalty.

## 4 Results

### 4.1 Demographic characteristics of Chinese older adults hospitalized for stroke

A total of 627 Chinese older adults hospitalized for stroke were recruited from three tertiary grade-A hospitals across two cities in China to participate in the survey. Among these participants, 509 (81.2%) were male and 118 (18.8%) were female. The age distribution was as follows: 199 (31.7%) participants were under 60, while 428 (68.3%) were 60 or older. Regarding nationality, 581 (92.6%) were Han, and 46 (7.4%) were from minority ethnic groups. Other demographic characteristics are presented in Table 1.

**Table 1:** Demographic characteristics among Chinese older adults hospitalized for stroke (*n* = 627).

Characteristics	*n*	%
Age (years)		
<60	199	31.7
≥60	428	68.3
Gender		
Male	509	81.2
Female	118	18.8
Nationality		
Han	581	92.6
Minority ethnic groups	46	7.4
Employment status		
Working	78	12.5
Retired	368	58.7
Unemployed	181	28.8
Marital status		
Unmarried	26	4.1
Married	511	81.5
Divorced	54	8.6
Widowed	36	5.8
Education level		
Primary school and below	67	10.7
Junior school	158	25.3
High school/secondary school	290	46.2
Junior college and above	112	17.8
Place of residence		
Cities	271	43.1
Towns	204	32.6
Rural area	152	24.3
Primary caregiver		
Parents	54	8.6
Spouse	455	72.5
Children	92	14.6
Others	26	4.3
Residence status		
Living alone	79	12.6
Living with others	548	87.4
Monthly income per capita (RMB)		
<1,000	70	11.2
1,000–<3,000	198	31.5
3,000–<5,000	256	40.8
≥5,000	103	16.5
Medical payment methods		
Urban employee medical insurance	227	36.2
Urban and rural residents medical insurance	386	61.5
Others	14	2.3
Stroke type		
Hemorrhagic stroke	205	32.7
Ischemic stroke	422	67.3
Duration of illness (months)		
<6	147	23.5
6–12	196	31.2
>12	284	45.3
Number of comorbidities		
None	11	1.7
1–3	452	72.1
>3	164	26.2
Number of stroke occurrences		
Once	375	59.8
Twice or more	252	40.2
Presence of functional impairment		
Yes	542	86.4
No	85	13.6
Activities of daily living		
Completely independent	79	12.6
Partially independent	498	79.5
Completely dependent	50	7.9

### 4.2 Scores of self-management behavior, family care, and chronic disease resource utilization

The total score of self-management behavior was (M = 165.94, SD = 51.26), with a mean score of (M = 3.32, SD = 0.96). Among the dimensions of SPSBAS, “safe medication management” had the highest average score (M = 4.18, SD = 0.95), while “disease management” had the lowest (M = 3.02, SD = 0.91). The average scores for the remaining dimensions were as follows: “emotional management” (M = 3.62, SD = 1.03), “activities of daily living management” (M = 3.41, SD = 0.96), “diet management” (M = 3.27, SD = 0.91), “social and interpersonal relationship management” (M = 3.12, SD = 0.98), and “rehabilitation exercise management” (M = 3.08, SD = 0.92).

The total score of family care was (M = 5.51, SD = 1.65), with an overall mean of (M = 1.10, SD = 0.63). Among the five dimensions of the F-APGARS, “adaptation” had the highest average score (M = 1.13, SD = 0.57), while “growth” had the lowest (M = 0.85, SD = 0.29). The average scores for the other dimensions were as follows: “affection” (M = 1.23, SD = 0.52), “partnership” (M = 1.16, SD = 0.47), and “respect” (M = 0.96, SD = 0.35).

The total score for chronic disease resource utilization was (M = 72.15, SD = 16.73), and the average score was (M = 3.28, SD = 0.86). Among the seven dimensions of the CIRS, “media and policy” had the highest average score (M = 4.02, SD = 1.05), while “family and friends” had the lowest (M = 2.51, SD = 0.76). The average scores for the other dimensions were as follows: “healthcare team” (M = 3.82, SD = 1.01), “organizational support” (M = 3.53, SD = 0.98), “personal coping” (M = 3.26, SD = 0.88), “community resources” (M = 3.12, SD = 0.83), and “work environment” (M = 2.75, SD = 0.79). The scores for the SPSBAS, F-APGARS, and CIRS are summarized in Table 2.

**Table 2:** The scores of SPSBAS, F-APGARS, and CIRS among Chinese older adults hospitalized for stroke [*n* = 627, M (SD)].

Dimensions	Number of items	Dimensional score		Average score of items		Ranking
		M	SD	M	SD	
SPSBAS total score	50	165.94	51.26	3.32	0.96	—
Disease management	11	33.22	10.53	3.02	0.91	7
Safe medication management	5	20.90	6.82	4.18	0.95	1
Diet management	8	26.16	9.15	3.27	0.91	4
Daily living management	8	27.28	9.02	3.41	0.96	3
Emotional management	5	18.10	5.73	3.62	1.03	2
Social and interpersonal management	6	18.72	5.61	3.12	0.98	5
Rehabilitation exercise management	7	21.56	7.23	3.08	0.92	6
F-APGARS total score	5	5.51	1.65	1.10	0.45	—
Adaptation	1	1.31	0.57	1.31	0.57	1
Partnership	1	1.16	0.47	1.16	0.47	3
Growth	1	0.85	0.29	0.85	0.29	5
Respect	1	0.96	0.35	0.96	0.35	4
Affection	1	1.23	0.52	1.23	0.52	2
CIRS total score	22	72.15	16.73	3.28	0.86	—
Healthcare team	3	11.46	3.15	3.82	1.01	2
Family and friends	3	7.53	2.39	2.51	0.76	7
Personal coping	3	9.78	2.86	3.26	0.88	4
Community resources	4	12.48	3.22	3.12	0.83	5
Media and policy	3	12.06	3.08	4.02	1.05	1
Organizational support	3	10.59	2.97	3.53	0.98	3
Work environment	3	8.25	2.45	2.75	0.79	6

### 4.3 Relationships between self-management behavior, family care, and chronic disease resource utilization

The total self-management behavior score was positively correlated with the total family care score (*r* = 0.615, *p* < 0.01), with significant positive correlations observed across all dimensions (*r* = 0.598–0.613, *p* < 0.01). Additionally, the total self-management behavior score was positively correlated with the total chronic disease resource utilization score (*r* = 0.536, *p* < 0.01), with positive correlations also observed across all dimensions (*r* = 0.515–0.553, *p* < 0.01), as shown in Table 3.

**Table 3:** The relationships between self-management behavior, family care, and chronic disease resource utilization among Chinese older adults hospitalized for stroke (*n* = 627, *r*).

Item	1	2	3
1 SPSBAS total score	—		
1.1 Disease management	0.908^**^		
1.2 Safe medication management	0.902^**^		
1.3 Diet management	0.896^**^		
1.4 Activity of daily living management	0.887^**^		
1.5 Emotional management	0.873^**^		
1.6 Social and interpersonal management	0.903^**^		
1.7 Rehabilitation exercise management	0.891^**^		
2 F-APGARS total score	0.615^**^	—	
2.1 Adaptation	0.613^**^	0.862^**^	
2.2 Partnership	0.608^**^	0.855^**^	
2.3 Growth	0.603^**^	0.845^**^	
2.4 Respect	0.598^**^	0.836^**^	
2.5 Affection	0.607^**^	0.819^**^	
3 CIRS total score	0.536^**^	0.557^**^	—
3.1 Healthcare team	0.539^**^	0.582^**^	0.873^**^
3.2 Family and friends	0.541^**^	0.573^**^	0.865^**^
3.3 Personal coping	0.553^**^	0.591^**^	0.832^**^
3.4 Community resources	0.522^**^	0.576^**^	0.847^**^
3.5 Media and policy	0.518^**^	0.556^**^	0.836^**^
3.6 Organizational support	0.515^**^	0.556^**^	0.853^**^
3.7 Work environment	0.527^**^	0.549^**^	0.851^**^

^**^p < 0.01 and —, *r* = 1.

### 4.4 Mediating effect of chronic disease resource utilization between self-management behavior and family care

**Figure 3:**
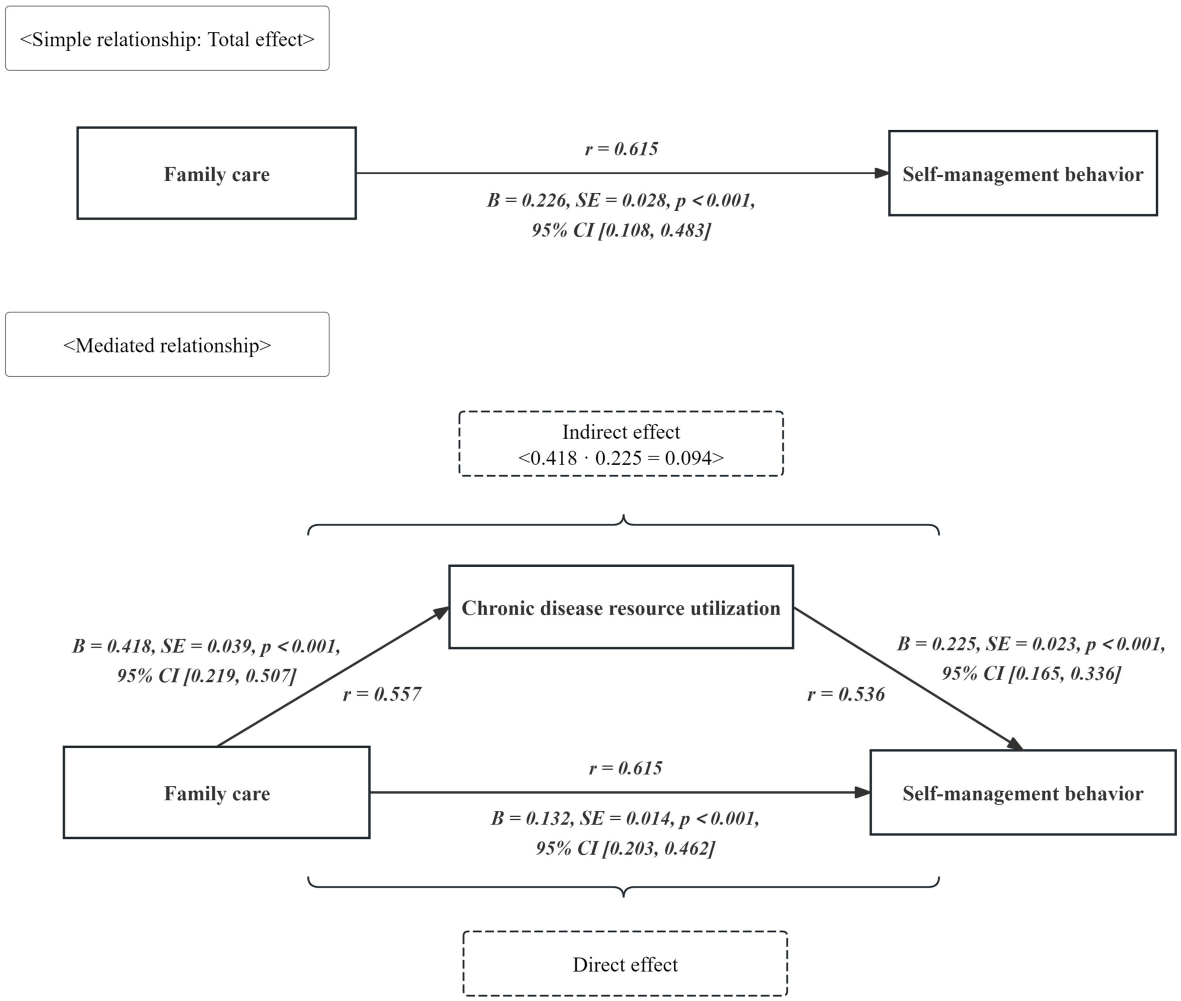
The direct, indirect, and total effects between the self-management behavior, family care, and chronic disease resource utilization.

The total impact of family care on self-management behavior was 0.226 (*p* < 0.01), with a 95 percent confidence interval (CI) of 0.108–0.483. The direct effect of family care on self-management behavior was 0.132 (*p* < 0.01), with a 95 percent CI of 0.203–0.462. The indirect impact of chronic disease resource utilization on self-management behavior was calculated as 0.418 × 0.225 = 0.094, accounting for 41.6% of the total effect value of 0.226 (*p* < 0.01). The bootstrapped CI ranged from 0.122 to 0.258, excluding 0, indicating statistical significance (*p* < 0.05), as shown in Table 4 and Figure 3.

**Table 4:** The mediating effect of chronic disease resource utilization between self-management behavior and family care among Chinese older adults hospitalized for stroke (*n* = 627).

Model pathways	Standardized effect (*B*)	SE	*t-*value	*p-*value	*F*	*R*	*R* ^2^	95 percent CI
Total effect					425.761	0.552	0.305	
Family care → Self-management behavior	0.226	0.028	8.071	<0.001^**^				[0.108, 0.483]
Direct effect					353.258	0.483	0.233	
Family care → Chronic disease resource utilization	0.418	0.039	10.718	<0.001^**^				[0.219, 0.507]
Family care → Self-management behavior	0.132	0.014	9.429	<0.001^**^				[0.203, 0.462]
Chronic disease resource utilization → Self-management behavior	0.225	0.023	9.783	<0.001^**^				[0.165, 0.336]
Indirect effect					—	—	—	
Family care → Chronic disease resource utilization → Self-management behavior	0.094	0.012	—	—				[0.122, 0.258]

^**^p < 0.01.

## 5 Discussion

### 5.1 Status of self-management behavior, family care, and chronic disease resource utilization

The self-management behavior score among the 627 Chinese older adults hospitalized for stroke was (M = 165.94, SD = 51.26), with an average item score of (M = 3.32, SD = 0.96), indicating a suboptimal level relative to established benchmarks. This intermediate level is concerning given the known association between self-management and functional recovery post-stroke (30). Compared to studies in western countries reporting higher self-management levels, these findings suggest a need for culturally adapted interventions in China. Also, this finding is consistent with the results of Guo et al. (31) in China. The observed level may be attributed to several factors, including the specialized knowledge required for rehabilitation and disease management. Many hospitalized older stroke survivors possess lower educational attainment, hindering their comprehension and acceptance of relevant health information. Consequently, this can lead to reduced adherence to treatment plans and challenges in implementing effective self-management. Furthermore, the extended treatment duration burdens patients, potentially affecting mood and diminishing motivation for self-management behaviors. Therefore, it is recommended that healthcare providers focus on enhancing treatment adherence and self-management efficacy through the following evidence-informed intervention strategies: first, implement personalized health education programs tailored to individual literacy and needs to build self-management competencies. Second, develop structured rehabilitation training plans emphasizing activities of daily living (ADL) training (6). Third, establish a comprehensive family-social support system incorporating regular follow-ups and mobile health technologies for sustained monitoring. Finally, prioritize psychological assessment and intervention, utilizing cognitive behavioral therapy (CBT) to alleviate anxiety and depressive symptoms (7). This integrated approach could significantly improve self-efficacy and adherence, thereby optimizing long-term functional outcomes and quality of life.

The total score for family care among the 627 Chinese older adults hospitalized for stroke was (M = 5.51, SD = 1.65), with an average item score of (M = 1.10, SD = 0.45), indicating a moderate level relative to the scale range. This observed level of family caregiving warrants clinical attention, given its well-established associations with critical health outcomes in chronic conditions, including prognosis and cognitive function in populations such as maintenance hemodialysis patients (32). Notably, the moderate level identified in this stroke cohort appears lower than family caregiving levels typically reported for other chronic disease groups, highlighting a specific vulnerability and unmet need among stroke patients in China. This finding is consistent with previous research by Luo (33) in the Chinese population. As stroke progresses, older adults often experience sensory, motor, and language impairments, significantly reducing their social engagement. This social withdrawal may contribute to feelings of loneliness and negative emotions, while the communication difficulties can strain interactions with family members, potentially diminishing perceived encouragement, assistance, and support. Consequently, satisfaction with family care may be reduced, contributing to the observed moderate level. Thus, the following evidence-based interventions are recommended to enhance family care quality: firstly, implement personalized family care training programs, emphasizing daily living assistance, complication prevention, and rehabilitation exercise techniques (13, 15). Secondly, establish a multidisciplinary follow-up team to provide continuous support through regular home visits and telemedicine guidance (17). Thirdly, develop a dedicated caregiver support platform to alleviate psychological distress and facilitate peer experience sharing (15). Lastly, compile standardized home care manuals incorporating visual aids to simplify operational procedures (18). These systematic interventions aim to address the identified gaps in family care, ultimately aiming to improve both caregiving quality and critical patient outcomes such as functional recovery, reduced complications, and enhanced quality of life.

The total score for chronic disease resource utilization among the 627 Chinese older adults hospitalized for stroke was (M = 72.15, SD = 16.73), with an average item score of (M = 3.28, SD = 0.86), indicating a moderate level of utilization. This finding has significant clinical implications as optimal resource utilization is strongly linked to improved prognosis and quality of life during stroke recovery, a relationship established across various chronic conditions including coronary heart disease (20). The observed moderate utilization in this stroke cohort is notably lower than levels reported in other chronic disease populations (20), and aligns with findings reported by He et al. (34) in elderly Chinese stroke inpatients. This pattern of suboptimal resource access may hinder recovery potential. Several factors likely contribute: these older adults often rely heavily on healthcare professionals for diagnosis, treatment, and daily management knowledge, limiting proactive resource seeking. Furthermore, constraints within China’s primary healthcare system restrict institutional resource availability for patients. Over time, the protracted treatment course can foster apathy, discouraging active pursuit of recovery resources and consequently lowering utilization rates. Compounding this, limited access to health education and information channels prevents many stroke patients from becoming aware of existing support resources. To address these barriers, we propose the following comprehensive intervention strategies: first, implement individualized health education programs to enhance awareness of chronic disease management resources among patients and caregivers. Second, leverage remote follow-up systems and mobile health technologies (e.g., specialized applications) to deliver self-management training and resource information (21). Additionally, establish a collaborative healthcare-community linkage mechanism to improve the referral process and integration of rehabilitation services and psychological support resources (19). Crucially, optimizing primary healthcare networks and providing targeted policy support are needed to address accessibility barriers, particularly for vulnerable populations, thereby systematically improving the utilization efficiency of chronic disease resources (22).

### 5.2 Positive correlations between self-management behavior, family care, and chronic disease resource utilization

A significant positive correlation was identified between self-management behavior and perceived family care among Chinese older adults hospitalized for stroke (*r* = 0.615, *p* < 0.01). This robust association underscores the critical role of family functioning in enabling patient self-management. Family care, conceptualized as satisfaction with family support and concern (7, 35), likely facilitates self-management by providing practical assistance with daily tasks, emotional encouragement, and reinforcement of health behaviors. Enhanced family care may bolster patients’ confidence and perceived self-efficacy in managing their condition. Consequently, interventions targeting family functioning improvement—such as structured caregiver training programs or family support groups—could be leveraged to directly enhance patient engagement in self-management behaviors. This perspective is supported by findings in related contexts (36).

Similarly, a significant positive correlation was found between self-management behavior and chronic disease resource utilization (*r* = 0.536, *p* < 0.01). This finding highlights the importance of accessible resources for effective self-management. Chronic disease resource utilization involves the acquisition and application of diverse social and healthcare resources to support disease management. Effective utilization of these resources—such as educational materials, community support services, or accessible healthcare professionals—can empower patients by providing necessary knowledge, skills, and social backing. This empowerment facilitates the adoption of positive coping strategies, which are known to promote self-management behaviors (19). Therefore, interventions designed to improve resource access and utilization—including personalized health education initiatives, strengthening primary healthcare networks with clear referral pathways, and implementing supportive policies—hold promise for directly augmenting patient self-management capabilities. This aligns with evidence suggesting the value of resource-enabling interventions (37–40).

### 5.3 Mediating role of chronic disease resource utilization between self-management behavior and family care

Bootstrap mediation analysis revealed that chronic disease resource utilization partially mediates the relationship between family care and self-management behaviors, with a significant indirect effect of 0.094 (*p* < 0.01), explaining 41.6% of the total effect. This partial mediation effect underscores a critical practical implication: family support alone is not sufficient for optimal self-management in stroke patients. When individuals perceive that family care falls short of their psychological expectations, they may struggle to fully mobilize their coping abilities and resources, potentially leading to compromised self-management (41). Chronic disease resource utilization serves as a vital physical and psychological protective factor, mitigating the adverse impact of insufficient family support and facilitating healthier behaviors and self-regulation. The recurrent nature of stroke not only challenges patients’ psychological coping abilities but also depletes their available coping resources (42, 43). Crucially, if patients cannot effectively access and utilize surrounding resources (e.g., healthcare services, community support), maladaptive coping and diminished self-management may ensue. Therefore, interventions must adopt a two-pronged approach: (1) strengthen the quality and responsiveness of family care to better meet patient needs and expectations, and (2) actively ensure stroke patients’ access to, and ability to mobilize, essential healthcare and community resources. Health education programs initiated during hospitalization are key to enhancing resource utilization; they can provide vital disease knowledge, bolster disease management skills, and empower patients to identify and leverage personal and external resources, thereby improving their capacity to cope with the disease (44).

### 5.4 Limitations

There are several limitations to this study. First, a convenience sampling method was employed, enrolling only 627 Chinese older adults hospitalized for stroke from three tertiary grade-A hospitals in two cities in China. This sampling approach may result in an unrepresentative sample and potentially biased, non-generalizable findings. Future research should address this limitation by using random, multi-center sampling with a larger sample size to capture a more representative and diverse population of older stroke patients. Additionally, the cross-sectional design is another limitation, which restricts the ability to establish causal relationships between self-management behavior, family care, and chronic disease resource utilization. Future research should use a longitudinal or prospective cohort design to better assess causal pathways and changes over time in these variables. Lastly, cultural expectations around filial piety may influence both the provision and perception of family care. This warrants exploration in future qualitative research.

## 6 Conclusion

This study found that self-management behavior, family care, and chronic disease resource utilization among the 627 Chinese older adults hospitalized for stroke were relatively moderate, indicating a need for improvement. Furthermore, significant positive correlations were found between self-management behavior, family care, and chronic disease resource utilization. Chronic disease resource utilization played a mediating role in the relationship between self-management behavior and family care. It is recommended that healthcare professionals enhance health education and provide psychological support for older adults hospitalized for stroke to improve family care and, ultimately, promote better self-management behavior.

## Data Availability

The original contributions presented in the study are included in the article/supplementary material, further inquiries can be directed to the corresponding authors.
